# Prevalence of cancer-related fatigue based on severity: a systematic review and meta-analysis

**DOI:** 10.1038/s41598-023-39046-0

**Published:** 2023-08-07

**Authors:** Ye-Eun Kang, Ji-Hae Yoon, Na-hyun Park, Yo-Chan Ahn, Eun-Jung Lee, Chang-Gue Son

**Affiliations:** 1 Research Center for CFS/ME, Daejeon Oriental Hospital of Daejeon University, Daejeon, Republic of Korea; 2 Department of Health Service Management, Daejeon University, Daejeon, Republic of Korea; 3 Department of Korean Rehabilitation Medicine, College of Korean Medicine, Daejeon University, Daejeon, Republic of Korea; 4 East-West Cancer Center of Daejeon Hospital, Daejeon University, Daejeon, Republic of Korea

**Keywords:** Cancer, Signs and symptoms

## Abstract

Cancer-related fatigue (CRF) affects therapeutic compliance and clinical outcomes including recurrence and mortality. This study aimed to comprehensively and comparatively assess the severity-based prevalence of CRF. From two public databases (PubMed and Cochrane Library), we extracted data containing information on both prevalence and severity of fatigue in cancer patients through December 2021. We conducted a meta-analysis to produce point estimates using random effects models. Subgroup analyses were used to assess the prevalence and severity by the organ/system tumor development, treatment phase, therapeutic type, sex and assessment method. A total of 151 data (57 studies, 34,310 participants, 11,805 males and 22,505 females) were selected, which indicated 43.0% (95% CI 39.2–47.2) of fatigue prevalence. The total CRF prevalence including ‘mild’ level of fatigue was 70.7% (95% CI 60.6–83.3 from 37 data). The prevalence of ‘severe’ fatigue significantly varied by organ/system types of cancer origin (highest in brain tumors 39.7% vs. lowest in gynecologic tumors 3.9%) and treatment phase likely 15.9% (95% CI 8.1–31.3) before treatment, 33.8% (95% CI 27.7–41.2) ongoing treatment, and 24.1% (95% CI 18.6–31.2) after treatment. Chemotherapy (33.1%) induced approximately 1.5-fold higher prevalence for ‘severe’ CRF than surgery (22.0%) and radiotherapy (24.2%). The self-reported data for ‘severe’ CRF was 20-fold higher than those assessed by physicians (23.6% vs. 1.6%). Female patients exhibited a 1.4-fold higher prevalence of ‘severe’ fatigue compared to males. The present data showed quantitative feature of the prevalence and severity of CRF based on the cancer- or treatment-related factors, sex, and perspective of patient versus physician. In the context of the medical impact of CRF, our results provide a comparative reference to oncologists or health care providers making patient-specific decision.

## Introduction

Cancer-related fatigue (CRF) refers to the subjective, persistent and distressing sense of tiredness or exhaustion related to cancer or cancer treatments^1^. CRF was the second most common complaint among 38 cancer-related symptoms, which followed pain in patients with advanced cancer^2^. Moreover, CRF is generally severe and not relieved by rest, unlike fatigue in sub-healthy individuals^3^. One study reported that fatigue rather than other 13 symptoms including pain and nausea/vomiting showed the most negative impact on quality of life (QoL) of breast cancer patients who completed primary therapy^1^.

In addition to reducing QoL, CRF affects the clinical outcomes of cancer patients as a dose-limiting side effect of therapeutics and can reduce the patient's willingness to undergo treatment, which impedes the opportune application or completion of treatment^1^. Moreover, the occurrence of CRF has been found to be associated with high mortality by predicting shorter recurrence-free survival (risk ratio 1.3; *p* < 0.01) and overall survival (risk ratio 1.2; *p* < 0.01) in breast cancer patients^1^. Another study also observed a shorter survival period in cancer patients with a higher fatigue level (risk ratio 1.2; *p* < 0.01)^1^. These facts imply that physicians have to assess the occurrence of CRF and its severity at regular intervals^1^.

Various pathophysiological mechanisms underlying CRF have been proposed, including activation of the proinflammatory cytokine network in the peripheral and central nervous systems^4,5^, dysregulation of cortisol release due to disturbance of the hypothalamic-pituitary adrenal (HPA) axis^6,7^, disruption in the circadian rhythm^8,9^, or metabolic exhaustion due to impaired adaptive response to energy depletion^10^. However, these hypotheses are still controversial, indicating that CRF might be a multifactorial disorder or linked to different contributors depending on individuals or cancer-associated conditions^11,12^. Some clinical trials have partially demonstrated positive outcomes in patients with CRF using agents such as dexamethasone or modafinil^13,14^ and nonpharmacological interventions, cognitive behavior therapy or exercise^15,16^. However, no mechanism-based standardized therapeutics for CRF exist to date^17,18^.

Exploring the severity-based prevalence of CRF and its related factors is essential for clinicians and researchers not only to manage cancer patients including evaluation of prognosis but also to identify suitable treatments^19^. Recently, two systematic reviews reported CRF prevalence of 49.0% and 52.0%, which partially but well investigated tumor type- and treatment-associated features of CRF^20,21^. These studies however lacked assessments of the severity-based CRF prevalence.

This systematic review and meta-analysis firstly aimed to investigate the comparative prevalence of CRF based on its severity across patients with different cancer types and who underwent different treatment phases.

## Methods

### Study design

To investigate comprehensively the prevalence of CRF based on its severity, studies from two public databases (PubMed and Cochrane Library) were systematically reviewed. We also performed a meta-analysis to produce the consistent and quantitative prevalence rate of CRF from individual data. This study was conducted in accordance with the PRISMA guidelines after registration in the PROSPERO (CRD42021270494).

### Data sources and eligibility criteria

A systematic literature search was conducted using two major electronic literature databases through December 31, 2021. We used the MeSH term “cancer-related fatigue”. In addition, we used the search terms ‘Fatigue [Title]’ AND ‘Prevalence [Title/Abstract]’ OR ‘Frequency [Title/Abstract]’ OR ‘Cancer [Title/Abstract]’, OR ‘Tumor [Title/Abstract]’ OR ‘Cancer-related fatigue [Title/Abstract]’ in PubMed, and ‘Fatigue [Record Title]’ AND ‘Prevalence [Title Abstract Keyword]’ and ‘Fatigue [Record Title]’ AND ‘Frequency [Title Abstract Keyword]’ in the Cochrane Library. Reports written in all languages were included.

Studies were included if they met the following criteria: (1) studies for patients with a history of cancer diagnosis, (2) studies with available diagnostic criteria for the identification of CRF and (3) studies with reports on the prevalence of CRF based on severity. Studies were excluded if they met the following criteria: (1) studies with nonhuman subjects; (2) studies containing no information on the prevalence or severity of CRF, (3) studies for which the articles did not have full text, (4) studies that used fewer than 20 participants or (5) review articles.

### Review process and data extraction

Three authors screened abstracts and articles using the inclusion/exclusion criteria. Full texts were reviewed, and data extraction was performed independently by the same three authors. The final selected articles were reexamined and cross-checked, and decisions were made by open discussion with corresponding author (Professor Son) in the case of disagreements. If necessary, the authors contacted the original authors via e-mail to obtain additional information or to resolve uncertainties.

The data were extracted from each study regarding the title, first author, publication year, study period, country, study design, participant demographic characteristics, type of fatigue assessment, type of tumor, status of cancer (cancer-free or not), treatment phase and its types, and prevalence and severity of fatigue. The collected data were coded as categorical variables in a coding book developed by our research team, and then arranged to compare the prevalence and severity of fatigue according to the types of tumors, assessment tools, interventions and the pretreatment or posttreatment status. The types of tumors were grouped into eight organs/systems: breast, gastrointestinal, urologic (including prostate and testis sites), lung, gynecologic (including ovary and cervix sites), hematologic, head and neck (including thyroid site), and brain (including WHO-defined grade I meningioma), respectively.

### Data classifications

The types of cancer treatments were categorized according to only what treatment patients received throughout their lifetime regardless of treatment phase. Patients with complete remission or no evidence of remaining tumors were classified as cancer-free patients. Patients exhibiting the presence of cancer were classified based on the following treatment phases: (1) ‘before’ those who had not yet started cancer treatments after being diagnosed with cancer; (2) ‘ongoing’ those who were undergoing cancer treatments for curative or palliative intent; and (3) ‘after’ those who exhibited the presence of cancer and were not undergoing cancer treatments after receiving previously certain therapy.

The severity of fatigue was categorized as ‘mild’, ‘moderate’, ‘severe’, and ‘moderate to severe’. Depending on the original data, a single level (‘severe’ or ‘moderate to severe’), 2 levels (‘moderate’ vs. ‘severe’ or ‘mild’ vs. ‘moderate to severe’), or 3 levels (‘mild’ vs. ‘moderate’ vs. ‘severe’) of fatigue severity were provided. Regarding the calculation of the prevalence of ‘moderate to severe’ fatigue, we used original data (presented as ‘moderate to severe’) and combined data in cases if ‘moderate’ and ‘severe’ fatigue data were provided as 2 levels (‘moderate’ vs. ‘severe’). We calculated the prevalence of ‘moderate to severe’ fatigue by combining the rates of them in cases where a study utilized a consistent CRF assessment tool within the same sample. We also estimated each severity-related fatigue prevalence according to the type of tumor and treatment-related factors mentioned above, using meta-analysis.

### Quality assessment

Two investigators independently assessed the quality of each included article using the Newcastle-Ottawa Scale (NOS), adapted for cross-sectional and cohort studies^22,23^. The assessment of each article's quality was based on three categories: participant selection, comparability of study controls, and appropriateness of outcome. An NOS score of 0–3 describes a study as low quality, a score of 4–6 indicates moderate quality, and a score of 7–9 denotes high quality. Any discrepancies in scoring were resolved through discussions among the four investigators.

### Meta-analysis

We conducted a meta-analysis to produce point estimates and 95% confidence intervals (CI) of the prevalence of CRF based on severity with subgroups. To improve the statistical analysis, the reported prevalence from each study was log-transformed, and pooled estimates were then converted back into the original prevalence scale. To account for the potentially high interstudy heterogeneity, the pooled outcome measures and their corresponding 95% CI were calculated using a random-effects model fitted with the restricted maximum likelihood estimator. The I^2^ statistic was used to evaluate the degree of heterogeneity between studies. All analyses were performed using the “meta” package (by Guido Schwarzer) in R version 4.2.1. Statistical significance was determined by a hypothesis test to analyze differences between the groups. In all analyses, *p* values of  < 0.05 indicated statistical significance.

## Results

### Characteristics of the included studies

**Figure 1:**
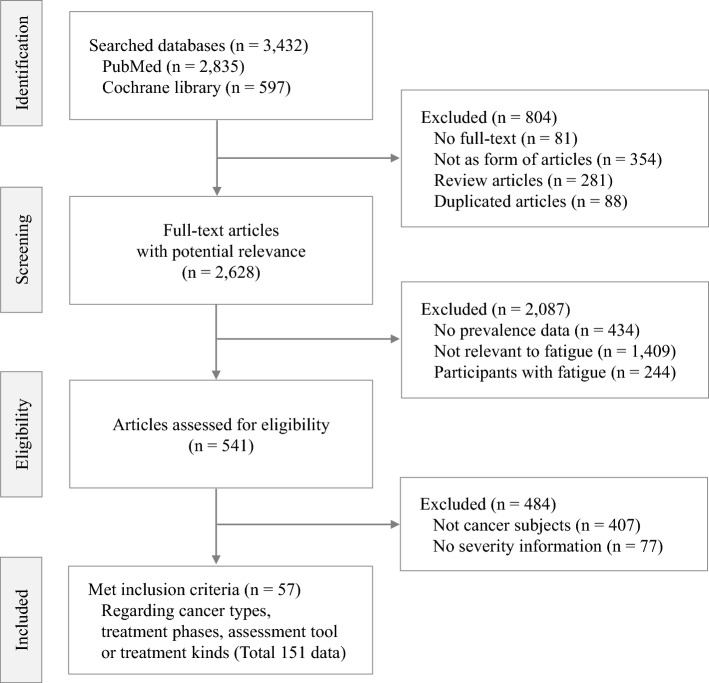
PRISMA flow-chart of patient selection for the meta-analysis.

Of the 3432 articles initially identified, 57 studies (38 cross-sectional and 19 cohort studies) finally fulfilled the eligibility criteria for this study (Fig. 1). According to the NOS scale, 29% (11 studies) and 71% (27 studies) of cross-sectional studies were classified as high and medium quality, respectively. For cohort studies, 32% (6 studies) were deemed high quality, while 68% (13 studies) were categorized as medium quality. These articles were conducted across 21 countries and enrolled a total of 34,310 participants (11,805 males and 22,505 females). All studies were published between 1999 and 2021 in English except 3 studies (Portuguese, German, and Spanish). A total of 151 multidimensional data regarding types of cancer, treatment phase, therapeutic types or evaluation tools were selected. Detailed characteristics of all included studies are provided in Table 1 and online (Table S5).

**Table 1:** Study characteristics.

Item	Number of studies (N. of data)	Number of participants (mean ± SD)			
		Male	Female	No information	Total
Total	57 (151)	11,805 (268 ± 407)	22,505 (417 ± 583)	–	34,310 (6,02 ± 869)^a^
Cross-sectional study	38 (99)	7651 (247 ± 334)	14,505 (403 ± 574)	–	22,156 (583 ± 804)
Longitudinal study	19 (52)	4154 (320 ± 557)	8000 (444 ± 617)	–	12,154 (640 ± 1010)
Severity classification^b^					
Single	25 (93)	9874	17,430	12,516	39,820
Binary	12 (21)	1908	3853	0	5761
Trinomial	23 (37)	4011	11,770	199	15,980
Type of cancer					
Breast	24 (30)	2	14,634	0	14,636
Urologic	12 (17)	2338	0	314	2652
Gastrointestinal	12 (14)	1019	888	1223	3130
Hematologic	10 (13)	2553	2190	1486	6229
Lung	7 (8)	907	614	797	2318
Gynecologic	4 (6)	0	3061	0	3061
Head and neck	3 (4)	138	194	151	483
Brain	3 (4)	30	88	355	473
Regardless of cancer type	23 (55)	6007	9086	13,486	28,579
Cancer status					
Cancer present	44 (109)	7263	16,637	13,461	37,361
Cancer-free	14 (26)	2542	5540	3424	11,506
Not defined	5 (16)	3928	7202	1564	12,694
Treatment phase					
Before treatment	11 (14)	1252	2286	65	3603
Ongoing treatment	22 (45)	3343	9007	4208	16,558
After treatment	11 (30)	1342	3556	6868	11,766
Type of treatment					
Chemotherapy	14 (15)	2203	3765	1780	7748
Surgery	11 (13)	240	3615	2551	6406
Radiotherapy	11 (13)	386	2471	1678	4535
Endocrine therapy	9 (10)	253	1418	1413	3084
Targeted therapy	3 (3)	0	0	202	202
Stem cell transplantation	1 (1)	57	41	0	98
Regardless of treatment type	30 (82)	10,030	17,282	8573	35,885
No treatment	11 (14)	1252	2286	65	3603
Assessment method					
Self-reported	56 (149)	15,793	30,725	12,715	59,233
Physician diagnosis	1 (2)	0	2328	0	2328
Assessment tool^c^					
BFI	17 (29)	2227	8804	145	11,176
CIS	7 (25)	384	594	465	1443
EORTC QLQ-C30	5 (23)	6966	10,534	4908	22,408
MDSAI	6 (22)	2202	4769	5593	12,564
MFI-20	6 (19)	1414	2086	1604	5104
FSS	4 (8)	316	186	0	502
PFS	4 (4)	85	1306	0	1391
Others (9 tools)^d^	9 (21)	2199	4774	0	6973
Continent					
Europe	25 (77)	10,666	15,110	5373	31,149
North America	13 (38)	2658	10,935	5738	19,331
Asia	12 (24)	1804	3730	1604	7138
Oceania	2 (5)	321	531	0	852
South America	3 (3)	146	315	0	461
Worldwide	2 (3)	176	2432	0	2608
Publication year					
Until 2010	16 (39)	5377	12,301	32	17,710
After 2010	41 (112)	10,416	20,752	12,683	43,851

^a^Total number of participants based on 50 studies, while sum of participants in subgroups is larger due to coming from each prevalence data.

^b^Single: severe, moderate to severe, strong; Binary: high/very high, moderate/severe, mild/moderate to intense, mild/moderate to severe; Trinomial: mild/moderate/severe, a little/quite a bit/very much, minor/moderate/severe, moderate/severe/very severe.

^c^CIS, Checklist Individual Strength; EORTC QLQ‐C30, European Organization for Research and Treatment of Cancer Quality of Life Questionnaire C30; BFI, Brief Fatigue Inventory; MDSAI, M. D. Anderson Symptom Inventory; MFI-20, Multidimensional Fatigue Inventory; FSS, Fatigue Severity Scale; PFS, Piper Fatigue Scale.

^d^CTCAE, Common Terminology Criteria for Adverse Event (ver. 3); National Comprehensive Cancer Network (NCCN) fatigue screening; RSCL, Rotterdam Symptom Checklist.

### Prevalence of CRF based on severity

**Figure 2:**
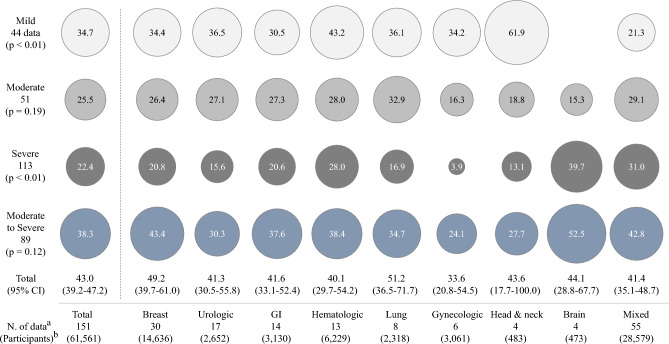
Prevalence of CRF according to severity and cancer type: The meta-analysis-derived prevalence of CRF according to 4 levels of severity and 8 organ/system types of cancer origin are presented inside each circle, and the prevalence rates are proportional to the size of the circles. The 95% CI is displayed only for the ‘total’ prevalence, which was synthesized with prevalence indicated as fatigue per data, regardless of severity. ^a^indicates the number of data used for meta-analysis. ^b^indicates the total number of patients enrolled for data analysis (some participants were counted repeatedly for mixed cancer). The detailed data can be found in Table S1.

The ‘total’ prevalence of CRF (regardless of severity from 151 data) was 43.0% (95% CI 39.2–47.2). Regarding the prevalence rates of CRF by the severity (but not mutually exclusive), the ‘mild’ was 34.7% (95% CI 31.3–38.6 from 44 data), ‘moderate’ 25.5% (95% CI 22.5–29.0 from 51 data), and ‘severe’ 22.4% (95% CI 19.3–25.9 from 113 data). The prevalence for ‘moderate to severe’ fatigue (from 89 data) was 38.3% (95% CI 34.8–42.2), respectively (Fig. 2; Table S1).

**Figure 3:**
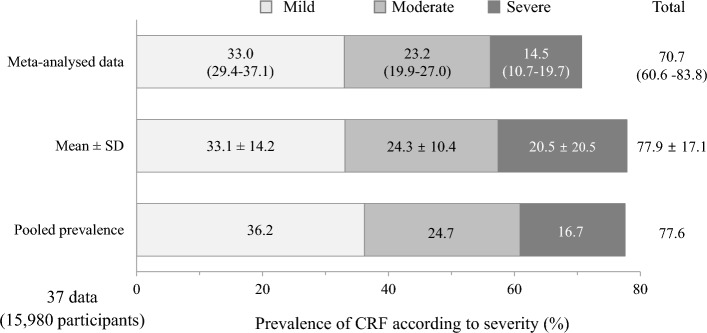
Prevalence rates of CRF according to severity using 37 data: The prevalence of CRF was presented in three levels, using only 37 data representing 'mild', 'moderate' and 'severe' CRF. Meta-analysis, mean estimate and pooled prevalence were provided for each fatigue severity and their sum.

When we analyzed 37 data (23 studies, total 15,980 participants) having information of 3-level fatigue severity within single study (Table 1), the ‘total’ prevalence was 70.7% (95% CI 60.6–83.8) composed of 33.0% for ‘mild’, 23.2% for ‘moderate’ and 14.5% for ‘severe’ CRF. The mean prevalence (77.9 ± 17.1% from 37 data) and pooled prevalence (77.6% from 15,980 participants) were a little higher than one by meta-analysis (Fig. 3).

### Prevalence of CRF by cancer-affected organ/system

Regarding the prevalence of CRF according to 8 types of organs/systems affected by cancer, patients with brain cancer reported the highest prevalence of ‘moderate to severe’ (52.5%) and ‘severe’ (39.7%), followed by patients with breast tumors, 43.4% ‘moderate to severe’ and 20.8% ‘severe’ fatigue, whereas the lowest prevalence of fatigue was reported in subjects with gynecologic cancer (24.1% of ‘moderate to severe’ and 3.9% of ‘severe’ fatigue). (Fig. 2; Table S1). The prevalence rates of ‘severe’ and ‘mild’ CRF were significantly different among patients with the 8 types of cancer (*p* < 0.01 for both severities).

### Prevalence of CRF by cancer treatment phase

**Figure 4:**
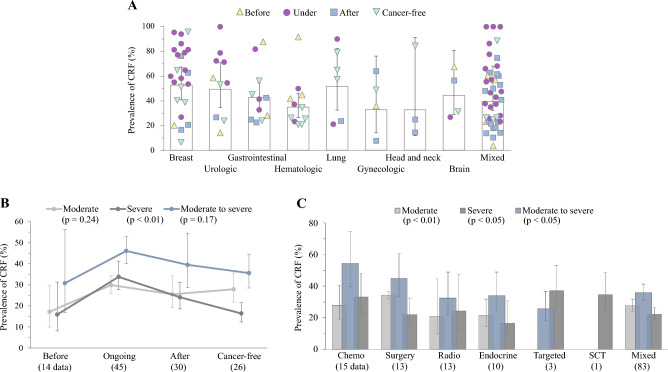
Prevalence rates of CRF according to phase and type of treatment: The meta-analysis-derived prevalence of CRF is presented by the organ/system of cancer origin using only data regarding the treatment phase (**A**), which showed statistical significance (*p* < 0.01). The prevalence of CRF in only those with ‘moderate’, ‘severe’ and ‘moderate to severe’ fatigue is presented by treatment phase (**B**) and type of treatment (**C**). The 95% CI is shown as a bar for each data. The statistical significance of the results in (**B**) and (**C**) is indicated by the *p* value. The detailed data can be found in Table S2.

When we analyzed CRF prevalence rates according to cancer treatment phase, they differed significantly, especially for ‘severe’ and ‘total’ fatigue (both *p* < 0.01). The high prevalence of CRF was observed in patients who were undergoing treatment across most cancer types (Fig. 4A). As expected, the lowest prevalence in patients before treatment (15.9% for ‘severe’ and 30.8% for ‘moderate to severe’) was drastically exchanged into the highest prevalence in patients undergoing treatment (33.8% for ‘severe’ and 46.1% ‘moderate to severe’), and then gradually decreased after treatment (24.1% for ‘severe’ and 39.5% ‘moderate to severe’). The patients with complete remission (referred to as ‘cancer-free’) presented overall similar CRF prevalence with those of before treatment (Fig. 4B; Table S2).

### Prevalence of CRF by treatment types

In the subgroup assessment of intervention-related differences, types of treatment significantly affected the prevalence of CRF at all severity levels (*p* < 0.01). The chemotherapy group exhibited the highest prevalence of ‘moderate to severe’ CRF (54.5%; 95% CI 39.7–74.7), followed by the surgery (45.0%) and endocrine therapy groups (34.0%), meanwhile the top three highest prevalence of ‘severe’ CRF were observed in those administered targeted therapy (37.1%), SCT (34.7%) and chemotherapy (33.1%). (Fig. 4C; Table S2).

### Prevalence of CRF by sex differences

**Figure 5:**
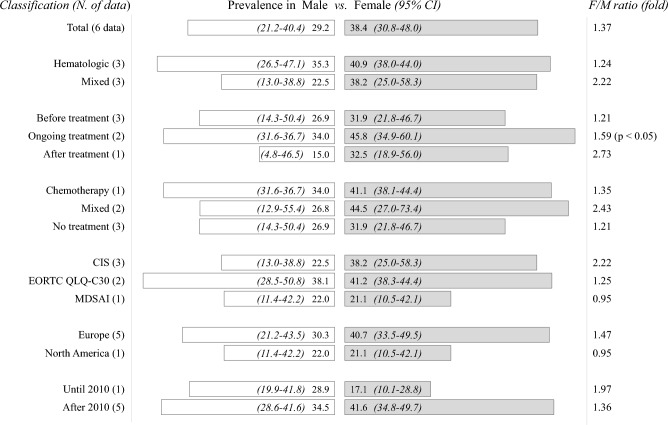
Prevalence of ‘severe’ CRF according to sex: The meta-analysis-derived prevalence of CRF is presented based on sex across each subclass, with only data with sex-related information shown. The statistical significance (*p* < 0.05) is shown in only the subgroup analysis for those who were receiving ongoing treatment. The detailed data can be found in Table S3.

To determine the effect of sex on the prevalence of CRF (only ‘severe’), we analyzed 6 data having for both males and females in same study. Female patients (38.4%; 95% CI 30.8–48.0) exhibited a 1.4-fold higher prevalence than males (29.2%; 95% CI 21.2–40.4), while this female-predominance was most obvious in patients after treatment (2.7-fold). The statistical significance was observed only in the subgroup that was undergoing treatment (1.6-fold, *p* < 0.05) (Fig. 5; Table S3).

### Prevalence of CRF by assessment methods and tools

**Figure 6:**
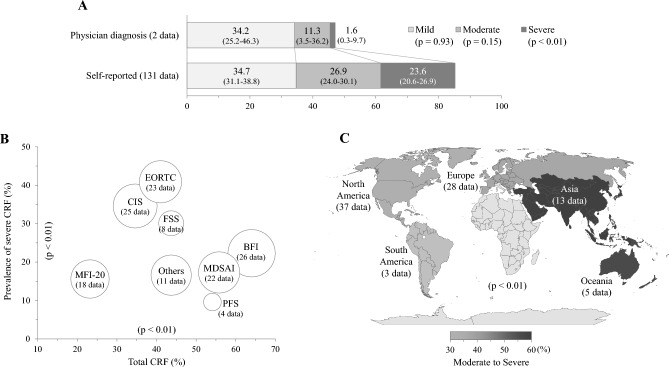
Prevalence of CRF according to assessment strategy and continent: The meta-analysis-derived prevalence of CRF was presented by assessment method (**A**), assessment tool (**B**) and continent (**C**). The detailed data including 95% CI can be found in Table S4.

The prevalence rates of CRF was drastically different based on whether the assessment method was self-reported or physician-directed, especially in the prevalence of ‘severe’ fatigue (23.6% vs. 1.6%; *p* < 0.01), while this gap was lower in assessments of ‘moderate’ and ‘mild’ CRF (Fig. 6A; Table S4).

A total of 16 different assessment tools were utilized to evaluate 151 data related to the prevalence of CRF, with 126 data (83.3%) being assessed using 7 specific tools. (Table 1). The prevalence of ‘severe’ fatigue was the highest in the data assessed by the EORTC QLQ‐C30, European Organization for Research and Treatment of Cancer Quality of Life Questionnaire C30 (EORTC, 40.9%) and Checklist Individual Strength (CIS, 34.6%) (*p* < 0.01), whereas total fatigue prevalence was the highest when measured by the Brief Fatigue Inventory (BFI, 64.0%) (*p* < 0.05, Fig. 6B; Table S4).

### Prevalence of CRF by continents and publication year

Regarding the countries conducted studies, the highest prevalence of ‘severe’ fatigue was observed in data obtained from Europe (27.5%), followed from Oceania (22.0%), whereas the CRF prevalence of ‘moderate to severe’ fatigue was highest data obtained from Asia (57.4%) (Fig. 6C; Table S4).

Prevalence of CRF was significantly different by publication year (divided by 2010), likely 33.1% versus 40.8% for 'moderate to severe' (*p* < 0.05), but was very similar as 21.9% versus 22.4% for 'severe' fatigue before and after 2010 (*p* = 0.90), respectively (Table S4).

## Discussion

In this study, we comparatively assessed the prevalence of CRF from 151 data (57 articles) composed of 34,310 cancer patients. Likely previous studies of 49.2% (from 129 data) and 52.0% (from 84 data)^20,21^, the overall average prevalence of 151 CRF data was determined to be 48.7% ± 25.1%. Furthermore, the pooled prevalence for a total of 34,310 patients was found to be 49.9% (data not shown). While meta-analysis result (43.0%, 95% CI 39.2–47.2) was slightly lower than that observed in two recent studies (Fig. 2), these gaps might be related to that one study did not consider severity^20^, and another contained partially severity-informed data (8 studies)^21^. In fact, when we analyzed 37 data containing 3 levels of severity (including ‘mild’ fatigue), the overall prevalence of CRF was 70.7% (95% CI 60.6–83.3) along with 77.6% of pooled prevalence for 15,980 participants (Fig. 3). In our study, approximately only 28% (44 data) of 151 data reflected ‘mild’ fatigue to total CRF prevalence (Table S1).

These results indicate that 4 to 7 of 10 cancer patients complain fatigue, contrary to one of 10 general population based on approximately 10.6%^24^. Fatigue is a nonspecific symptom not only in diseased population but also in healthy subjects, and then the medical impact of fatigue relies on its severity and/or duration^25^. Accordingly, the present study stressed the severity-based prevalence of CRF. Fatigue severity directly affects QoL or clinical outcomes including survival in individuals with disorders, for example, advanced kidney disease or various tumors^26–28^. In general, a fatigue level of ‘moderate to severe’ is considered clinically significant^29,30^. Based on our results, we found that approximately 40% of cancer patients (38.3% from 151 data, 47.7% from 37 data) are suffering from 'moderate to severe' fatigue (Figs. 2 and 3). This CRF severity however is a little milder than 48.5% of ‘severe’ fatigue among patients with chronic obstructive pulmonary disease (COPD) that was known well as a fatigue-causing representative disease^29,30^.

The prevalence of CRF and its severity are generally affected by disease status, clinical stage, the invaded organ/system, and/or treatment state^31,32^, which was also observed in our results (Figs. 2 and 4A–C). Regarding CRF prevalence and severity by treatment phase, the treatment itself raised twofold of ‘severe’ CRF prevalence (33.8%) compared to before treatment (15.9%), and it declined moderately after treatment (24.1%) (Fig. 4A and B; Table S2). Chemotherapy (33.1%) was associated with the highest prevalence of ‘severe’ CRF comparing to 2 other conventional cancer treatments: surgery (22.0%) and radiotherapy (24.2%) (Fig. 4C; Table S2). One longitudinal study of patients with colorectal cancer showed a continuous increase in the prevalence of CRF during chemotherapy^31,32^. Contrary to the conventional chemotherapy, the targeted therapy induces less damage to normal cells^31,32^. Then the prevalence of ‘severe’ fatigue among patients treated with targeted therapy (37.1%) (Fig. 4C) would be associated with the characteristics of enrolled patients (advanced and incurable solid tumors)^31,32^ (Table S5). SCT-related high prevalence (34.7%) of ‘severe’ CRF was observed even among patients with complete remission in our data^31,32^ (Fig. 4C, Table S5). This is related to the fact that SCT itself is a highly aggressive intervention, as a recent study reported a 40.5% of ‘severe’ fatigue in patients after SCT treatment^31,32^.

On the other hand, the significant difference in CRF prevalence rates (especially for ‘severe’) among patients with the 8 types of cancer was similarly repeated even though we removed the data of patients undergoing treatment (Fig. 2; Suppl. Fig. 1). Unlike the ‘moderate to severe’ prevalence (from 24.1% in gynecologic cancer to 52.5% in brain tumors), the prevalence of ‘severe’ fatigue was significantly different by approximately tenfold (39.7% in brain tumors vs. 3.9% in gynecologic cancer) (Fig. 2; Table S1). This high prevalence of fatigue observed in patients with brain tumors could be brain-specific. Structural and functional changes in the brain are known to be the pathological mechanisms underlying the high prevalence of ‘severe’ fatigue observed in patients with neurological disease, such as 64% in patients with Parkinson's disease^33^, 74% in patients with multiple sclerosis^34^, and 69% in patients with spinocerebellar ataxia^35^.

Besides malignant biological processes associated with tumors and/or treatment-related adverse effects, common medical comorbidities, such as insomnia or pain, as well as psychological factors including depression anxiety, or fear regarding death or recurrence can cause CRF^36–38^. As our results showing a 1.4-fold higher prevalence of CRF in females (Fig. 5; Table S3), females have been known to exhibit approximately 1.5-fold higher prevalence of myalgic encephalomyelitis/chronic fatigue syndrome (ME/CFS), a representative fatigue-specific disease^39^. Female cancer patients suffer more from psychiatric disorders, including distress, anxiety, and depression^40,41^. Furthermore, female patients are generally more susceptible to anticancer drugs due to sex-related disparities in the pharmacokinetic profile, which are responsible for the ∼20% overexposure^42^. For example, 5-fluorouracil resulted in up to 26% higher exposure levels in females^43^, and temozolomide had a 19% lower clearance rate in females than in males^44^. Ethnicity is also known to affect the prevalence and degree of fatigue^45^, then studies from Asian countries showed an almost 1.8-fold higher prevalence of ‘moderate to severe’ CRF (57.4%) than studies from South America (31.9%) (Fig. 6C; Table S4).

CRF profoundly affects treatment compliance and outcomes; thus, both cancer patients and healthcare providers endorse the benefits of routine assessments of fatigue^46,47^. The American Society of Clinical Oncology (ASCO) and the National Comprehensive Cancer Network (NCCN) have also published a Clinical Practice Guideline that recommends clinicians to regularly conduct screenings for CRF^1,48^. In our results, the physician-diagnosed prevalence of ‘severe’ CRF was only 1.6%; unlike the 23.6% observed in self-reported CRF. The prevalence gap, however, narrowed in the ‘moderate’ fatigue (11.3% vs. 26.9%) and was reversed in the prevalence of ‘mild’ fatigue (34.2% vs. 34.7%) (Fig. 6A; Table S4). Physicians may underestimate the importance of fatigue, which leads to the notable presence of barriers to the communication of patients’ fatigue. On study reported that an estimated 66% of cancer patients had never spoken to their doctor about fatigue^49^. The lower prevalence of physician-diagnosed CRF, as compared to self-reported CRF, may be attributed to the potential lack of implementation of the CRF clinical practice guideline in practice and/or the discrepancy in assessment tools. Because there is no objective biomarker to measure fatigue level, the CRF are commonly assessed via patient-reported questionnaires^50^. In present study, total 16 kinds of assessment tools were employed, with the most frequently application of BFI. The choice of assessment tools affects estimates of the prevalence of CRF; for example, an approximately 2.7-fold gap in the ‘severe’ CRF prevalence between data based on the BFI and MDSAI (32.0% vs. 12.0%) among disease-free survivors of breast cancer^30,51^. The different cutoff points also affected the CRF prevalence, for example, among the our data used EORTC QLQ-C30 tool, the judged scores for ‘severe’ fatigue varied likely over 50 points^52^, 45 points^53^, and 40 points^54,55^, respectively. These results indicate the importance of proper choice of assessment tools and cutoff pints for clinical severity of CRF.

Above the different cutoff values used in assessments of CRF and severity is one of limitations of our study, along with the considerable heterogeneity in patient populations and questionnaires used. Fatigue generally includes physical, mental, emotional and/or cognitive fatigue; however, we could not extract those data in our assessments of CRF. Although the clinical stage is a crucial factor affecting prevalence and severity of CRF, the present study also had no data regarding disease stage subgroups due to the lack of those data. Some subset analyses were based on a small number of cases, which raises the possibility of random variation.

Despite the limitations above, our study provided a severity-based feature of CRF from the aspect of cancer-originated organ/system, treatment phase and intervention types. In the context of the medical impact of fatigue in cancer patients, the current results provide a comparative reference for patient-specific decision making performed by oncologists or health care providers.

## Data availability

All data analyzed during this study are are included in this published article and supplementary information files.

### Supplementary Information

Supplementary Information.

Supplementary Legends.

Supplementary Figure 1.

Supplementary Figure 2.
