# Supplementary material for: Prevalence of cancer-related fatigue based on severity: a systematic review and meta-analysis
Source: Sci Rep. 2023 Aug 7;13:12815. doi: 10.1038/s41598-023-39046-0 (PMC10406927; doi:10.1038/s41598-023-39046-0)
Supplement: Supplementary file 1 — Supplementary Information. [file 41598_2023_39046_MOESM1_ESM.docx]

| **Table S1.**  Prevalence of CRF based on severity assessed by cancer type. | | | | | |
| --- | --- | --- | --- | --- | --- |
| **Type of cancer** | **Prevalence, % (95% CI; number of data)** | | | | |
|  | **Mild** | **Moderate** | **Severe** | **Moderate to severe^a^** | **Total^b^** |
| Total prevalence | 34.7 (31.3-38.6)  (44) | 25.5 (22.5-29.0) (51) | 22.4 (19.3-25.9) (113) | 38.3 (34.8-42.2) (89) | 43.0 (39.2-47.2)  (151) |
| Breast | 34.4 (29.6-40.1) (11) | 26.4 (20.5-33.9) (12) | 20.8 (15.0-28.9) (21) | 43.4 (36.4-51.7) (21) | 49.2 (39.7-61.0) (30) |
| Urologic | 36.5 (31.0-43.0) (9) | 27.1 (23.1-31.9) (8) | 15.6 (12.2-20.0) (13) | 30.3 (24.4-37.6) (12) | 41.3 (30.5-55.8) (17) |
| Gastrointestinal | 30.5 (25.8-36.1) (4) | 27.3 (20.8-35.9) (5) | 20.6 (14.5-29.3) (12) | 37.6 (30.4-46.6) (7) | 41.6 (33.1-52.4) (14) |
| Hematologic | 43.2 (24.7-75.7) (3) | 28.0 (20.3-38.7) (3) | 28.0 (22.3-35.2) (11) | 38.4 (28.4-51.8) (5) | 40.1 (29.7-54.2) (13) |
| Lung | 36.1 (31.9-40.8) (4) | 32.9 (27.0-40.0) (3) | 16.9 (7.1-40.0)  (5) | 34.7 (23.5-51.3) (6) | 51.2 (36.5-71.7) (8) |
| Gynecologic | 34.2 (25.2-46.3)  (2) | 16.3 (6.0-44.4)  (3) | 3.9 (1.1-14.4)  (4) | 24.1 (12.4-46.9)  (5) | 33.6 (20.8-54.5) (6) |
| Head and neck | 61.9 (54.0-71.0) (2) | 18.8 (6.7-52.7)  (2) | 13.1 (7.6-22.6)  (4) | 27.7 (12.1-63.1) (2) | 43.6 (17.7-100.0) (4) |
| Brain | - | 15.3 (9.7-24.3)  (2) | 39.7 (28.4-55.6) (3) | 52.5 (34.2-80.4) (3) | 44.1 (28.8-67.7) (4) |
| Mixed | 21.3 (12.3-36.9) (9) | 29.1 (22.5-37.6) (13) | 31.0 (24.9-38.7) (40) | 42.8 (35.9-50.9) (28) | 41.4 (35.1-48.7) (55) |
| ^a^ This value were calculated using the prevalence data described as ‘moderate to severe’ or by combined ‘moderate’ and ‘severe’ fatigue prevalence together.  ^b^ Irrespective of severity, prevalence rates labeled fatigue per data are synthesized. Total number of data may exceed sum of each data for severity, due to not mutually exclusive. | | | | | |

| **Table S2.** Prevalence and severity of CRF by cancer treatment phase and types. | | | | | |
| --- | --- | --- | --- | --- | --- |
| **Treatments** | **Prevalence (95% CI; number of data)** | | | | |
|  | **Mild** | **Moderate** | **Severe** | **Moderate to**  **severe^a^** | **Total^b^** |
| **Cancer status** |  |  |  |  |  |
| Cancer present | 34.8 (31.3-38.8) (29) | 23.5 (19.5-28.2) (28) | 25.0 (20.9-30.0) (78) | 39.6 (34.4-45.6) (51) | 43.3 (38.4-48.8) (101) |
| Cancer-free | 31.8 (23.0-44.0) (9) | 27.9 (21.8-35.6) (12) | 16.4 (12.4-21.5) (23) | 35.6 (28.5-44.4) (16) | 38.8 (30.9-48.7) (26) |
| **Treatment phase** |  |  |  |  |  |
| Before treatment | 36.5 (27.0-49.4) (5) | 17.2 (9.9-29.8) (6) | 15.9 (8.1-31.3) (12) | 30.8 (16.8-56.3) (7) | 36.4 (23.4-56.8) (14) |
| Ongoing treatment | 34.1 (29.4-39.4) (18) | 29.9 (26.0-34.5) (18) | 33.8 (27.7-41.2) (30) | 46.1 (40.0-53.1) (33) | 57.8 (51.1-65.4) (45) |
| After treatment | 40.6 (37.2-44.3) (2) | 25.5 (19.1-34.2) (5) | 24.1 (18.6-31.2) (30) | 39.5 (28.7-54.5) (5) | 32.1 (25.8-40.0) (30) |
| **Type of treatment** |  |  |  |  |  |
| Chemotherapy | 31.4 (23.6-41.7) (6) | 27.8 (19.1-40.5) (7) | 33.1 (22.8-48.1) (12) | 54.5 (39.7-74.7)  (8) | 57.2 (43.8-74.6) (15) |
| Surgery | 41.1 (27.9-60.5) (3) | 34.2 (31.9-36.6) (6) | 22.0 (14.9-32.4) (11) | 45.0 (33.5-60.6) (8) | 48.3 (36.8-63.4) (13) |
| Radiotherapy | 48.1 (40.2-57.4) (6) | 21.0 (9.9-44.5)  (3) | 24.2 (12.3-47.3) (9) | 32.5 (21.6-48.9) (7) | 56.0 (43.2-72.6) (13) |
| Endocrine therapy | 37.5 (30.2-46.5) (6) | 21.5 (14.5-31.8) (6) | 16.5 (8.9-30.6) (9) | 34.0 (23.7-48.9) (7) | 60.4 (48.2-75.8) (10) |
| Targeted therapy | - | - | 37.1 (26.0-53.1) (2) | 25.8 (18.2-36.7) (1) | 31.7 (23.3-43.1) (3) |
| Stem cell  transplantation | - | - | 34.7 (24.8-48.6) (1) | - | 34.7 (24.8-48.6) (1) |
| Mixed | 30.1 (25.4-35.7) (18) | 27.7 (24.1-31.8) (24) | 22.2 (18.6-26.5) (58) | 35.9 (31.2-41.3) (31) | 38.8 (34.4-43.9) (83) |
| ^a^ This value were calculated using the prevalence data described as ‘moderate to severe’ or by combined ‘moderate’ fatigue and ‘severe’ fatigue prevalence together.  ^b^ Irrespective of severity, prevalence rates labeled fatigue per data are synthesized. Total number of data may exceed sum of each data for severity, due to not mutually exclusive. | | | | | |

| **Table S3.**  Prevalence of CRF by sex differences. | | | |
| --- | --- | --- | --- |
| **Items** | **Prevalence of severe fatigue (95% CI)** | | |
|  | **Male** | **Female** | **Total** |
| **Total (6 data)** | 29.2 (21.2-40.4) | 38.4 (30.8-48.0) | 32.4 (24.4-43.0) |
| **Type of cancer** |  |  |  |
| Hematologic (3 data) | 35.3 (26.5-47.1) | 40.9 (38.0-44.0) | 34.9 (24.3-50.0) |
| Mixed (3 data) | 22.5 (13.0-38.8) | 38.2 (25.0-58.3) | 29.6 (18.0-48.8) |
| **Treatment phase** |  |  |  |
| Before treatment (3 data) | 26.9 (14.3-50.4) | 31.9 (21.8-46.7) | 29.1 (18.1-46.6) |
| Ongoing treatment (2 data) | 34.0 (31.6-36.7) | 45.8 (34.9-60.1) | 40.4 (32.6-49.9) |
| After treatment (1 data) | 15.0 (4.8-46.5) | 32.5 (18.9-56.0) | 21.7 (12.6-37.3) |
| **Type of treatment** |  |  |  |
| Chemotherapy (1 data) | 34.0 (31.6-36.7) | 41.1 (38.1-44.4) | 37.3 (35.3-39.3) |
| Mixed (2 data) | 26.8 (12.9-55.4) | 44.5 (27.0-73.4) | 33.1 (15.7-70.0) |
| No treatment (3 data) | 26.9 (14.3-50.4) | 31.9 (21.8-46.7) | 29.1 (18.1-46.6) |
| **Assessment tool** |  |  |  |
| CIS (3 data) | 22.5 (13.0-38.8) | 38.2 (25.0-58.3) | 29.6 (18.0-48.8) |
| EORTC QLQ-C30 (2 data) | 38.1 (28.5-50.8) | 41.2 (38.3-44.4) | 39.4 (33.3-46.7) |
| MDSAI (1 data) | 22.0 (11.4-42.2) | 21.1 (10.5-42.1) | 21.5 (13.4-34.6) |
| **Continent** |  |  |  |
| Europe (5 data) | 30.3 (21.2-43.5) | 40.7 (33.5-49.5) | 34.6 (25.9-46.1) |
| North America (1 data) | 22.0 (11.4-42.2) | 21.1 (10.5-42.1) | 21.5 (13.4-34.6) |
| **Publication year** |  |  |  |
| Until 2010 (1 data) | 28.9 (19.9-41.8) | 17.1 (10.1-28.8) | 23.5 (17.3-31.8) |
| After 2010 (5 data) | 34.5 (28.6-41.6) | 41.6 (34.8-49.7) | 34.8 (25.9-46.7) |

| **Table S4.** Prevalence and severity of CRF by assessment methods, continents and publication year. | | | | | |
| --- | --- | --- | --- | --- | --- |
| **Items** | **Prevalence (95% CI; number of data)** | | | | |
|  | **Mild** | **Moderate** | **Severe** | **Moderate to severe^a^** | **Total^b^** |
| **Assessment method** |  |  |  |  |  |
| Self-reported | 34.7 (31.1-38.8) (42) | 26.9 (24.0-30.1) (49) | 23.6 (20.6-26.9) (111) | 39.4 (36.1-43.1) (87) | 42.9 (39.0-47.2) (149) |
| Physician diagnosis | 34.2 (25.2-46.3) (2) | 11.3 (3.5-36.2) (2) | 1.6 (0.3-9.7)  (2) | 12.9 (3.7-44.8)  (2) | 48.1 (27.3-84.6)  (2) |
| **Assessment tool** |  |  |  |  |  |
| BFI | 32.0 (23.8-43.1) (16) | 26.8 (21.1-34.1) (16) | 22.3 (14.2-35.1) (18) | 43.2 (35.1-53.1) (27) | 64.0 (54.6-74.9) (29) |
| CIS | - | - | 34.6 (29.4-40.6) (25) | - | 34.6 (29.4-40.6) (25) |
| EORTC QLQ-C30 | 57.2 (47.2-69.3) (2) | 23.7 (22.0-25.5) (1) | 40.9 (27.9-59.8) (10) | 31.6 (27.6-36.1) (14) | 41.0 (34.6-48.6) (23) |
| MDSAI | 36.8 (33.2-40.9) (13) | 23.7 (20.1-28.0) (10) | 17.3 (12.8-23.4) (10) | 35.5 (30.4-41.4) (22) | 55.8 (46.3-67.2) (22) |
| MFI-20 | 19.6 (16.6-23.2)  (1) | 25.2 (16.8-37.9) (7) | 15.6 (11.7-20.9) (19) | 47.8 (39.4-57.9) (7) | 23.2 (16.1-33.3) (19) |
| FSS | 28.6 (20.3-40.2)  (2) | 21.0 (10.1-43.8) (2) | 30.1 (17.7-51.2) (8) | 36.7 (22.0-61.2) (2) | 43.7 (27.8-68.8) (8) |
| PFS | 25.7 (20.9-31.8)  (2) | 35.3 (21.1-59.1)  (3) | 9.6 (3.5-26.3)  (3) | 38.7 (22.5-66.6)  (4) | 54.1 (40.2-72.9)  (4) |
| Others | 33.8 (29.5-38.8) (8) | 24.8 (18.3-33.6)  (12) | 16.6 (11.3-24.5)  (20) | 37.6 (26.5-53.4) (13) | 43.7 (34.9-54.8) (21) |
| **Continent** |  |  |  |  |  |
| Europe | 37.4 (29.1-48.1) (9) | 24.8 (19.9-30.9) (16) | 27.5 (23.5-32.1) (65) | 36.8 (32.5-41.7) (28) | 38.7 (34.6-43.3) (77) |
| North America | 35.8 (31.6-40.6) (17) | 26.8 (23.9-30.2) (14) | 17.9 (13.1-24.5) (15) | 35.9 (31.7-40.6) (37) | 50.2 (43.4-58.0) (38) |
| Asia | 24.8 (16.2-38.0) (11) | 25.3 (17.3-36.9) (12) | 20.3 (14.2-29.0) (24) | 57.4 (42.2-78.0) (13) | 39.7 (27.5-57.5) (25) |
| Oceania | 32.9 (27.9-38.7) (1) | 35.5 (31.7-39.8) (5) | 22.0 (15.9-30.4) (5) | 55.5 (50.7-60.7) (5) | 64.2 (51.9-79.3) (5) |
| South America | 32.1 (20.4-50.6) (3) | 28.9 (17.6-47.3) (2) | 7.7 (2.9-20.7)  (2) | 31.9 (20.5-49.6) (3) | 63.9 (41.2-99.0) (3) |
| **Publication year** |  |  |  |  |  |
| Until 2010 | 40.6 (35.6-46.3) (12) | 24.0 (18.5-31.3) (10) | 21.9 (16.1-29.9) (23) | 33.1 (28.9-37.9) (26) | 43.1 (36.6-50.6) (39) |
| After 2010 | 32.6 (28.4-37.4) (32) | 25.9 (22.4-30.0) (41) | 22.4 (19.0-26.5) (90) | 40.8 (36.1-46.2) (63) | 43.0 (38.4-48.2) (112) |
| ^a^ This value were calculated using the prevalence data described as ‘moderate to severe’ or by combined ‘moderate’ fatigue and ‘severe’ fatigue prevalence together.  ^b^ Irrespective of severity, prevalence rates labeled fatigue per data are synthesized. Total number of data may exceed sum of each data for severity, due to not mutually exclusive. | | | | | |

| **Table S5.** Summary lists of 57 studies for characteristics of CRF. | | | | | | | |
| --- | --- | --- | --- | --- | --- | --- | --- |
| **1^ST^ author**  **(Year; Ref.)** | **Kind of tumors** | **Number of participants**  **(male/female)** | **Treatment** | | **Fatigue** | | |
|  |  |  | **Phase** | **Kind** | | **Assessment** | **Levels^a^** |
| Stone P et al  (1999; ^1^) | Mixed | 95  (41/54) | After | Mixed | | FSS | 1 |
| Stone P et al  (2000; ^2^) | Breast, Urologic, Lung, Mixed | 227  (127/100) | Present | Mixed | | FSS | 1 |
| Stone P et al  (2000; ^3^) | Urologic | 58  (58/0) | Before, Ongoing | Endocrine, Mixed | | FSS | 3 |
| Stone P et al  (2001; ^4^) | Mixed | 64  (32/32) | Ongoing | Radio | | FSS | 1 |
| Wang XS et al  (2002; ^5^) | Hematologic | 228  (55/173) | Ongoing | Mixed | | BFI | 1 |
| de Jong N et al  (2004; ^6^) | Breast | 152  (0/152) | Ongoing, After | Chemo | | Others | 3 |
| Hickok JT et al  (2005; ^7^) | Mixed, Urologic, Breast | 372  (166/206) | Before, Ongoing | Radio | | MDSAI | 2 |
| Meeske K et al  (2007; ^8^) | Breast | 800  (0/800) | Free | Mixed | | PFS | 2 |
| Storey DJ et al  (2007; ^9^) | Mixed, Breast, GI, Urologic, Gynecologic | 2867  (1030/1837) | Mixed, Ongoing, Free, Present | Mixed, Endocrine | | EORTC QLQ-C30 | 3, 1 |
| Gielissen MF et al  (2007; ^10^) | Hematologic | 98  (57/41) | Free | SCT | | CIS | 1 |
| Servaes P et al  (2007; ^11^) | Breast | 121  (0/121) | Free | Mixed | | CIS | 1 |
| Kim SH et al  (2008; ^12^) | Breast | 1884  (0/1884) | Free | Surgery | | BFI | 3 |
| Goedendorp MM et al  (2008; ^13^) | Urologic, Breast, GI | 179  (82/97) | Before | None | | CIS | 1 |
| Kuhnt S et al  (2009; ^14^) | Mixed | 646  (280/366) | After | Mixed | | MFI-20 | 2 |
| Huang X et al  (2010; ^15^) | Breast | 315  (0/315) | Present | Endocrine | | BFI | 3 |
| Prigozin A et al  (2010; ^16^) | Breast | 51  (0/51) | Ongoing | Chemo | | MDSAI | 3 |
| de Araújo Lamino D et al (2011; ^17^) | Breast | 182  (0/182) | Present | Mixed | | PFS | 2 |
| Hung R et al  (2011; ^18^) | Lung | 350  (128/222) | Free | Surgery | | BFI | 2 |
| Liu Y et al  (2011; ^19^) | Mixed | 256  (151/105) | Present | Mixed | | MDSAI | 1 |
| Li Y et al  (2011; ^20^) | Breast | 252  (0/252) | Ongoing | Chemo | | PFS | 2 |
| Karthikeyan G et al  (2012; ^21^) | Mixed | 121  (63/58) | Ongoing | Radio, Chemo, Mixed | | BFI | 3 |
| Mota DD et al  (2012; ^22^) | GI | 157  (85/72) | Present | Mixed | | PFS | 3 |
| Goedendorp MM et al  (2013; ^23^) | Breast, Mixed | 60  (20/40) | After | Mixed, Surgery, Radio, Endocrine | | CIS | 1 |
| Tian J et al  (2013; ^24^) | Mixed, Head and neck, Lung, GI, Breast, Gynecologic | 715  (378/337) | Before, After | Mixed, Radio, Surgery, Chemo | | MFI-20 | 1 |
| Cleeland CS et al  (2013; ^25^) | Mixed, Breast, GI, Urologic, Lung | 3106  (936/2170) | Mixed, Ongoing, Free, Present | Mixed | | MDSAI | 1 |
| Wang XS et al  (2014; ^26^) | Breast, GI, Urologic, Lung | 2692  (709/1983) | Free, Ongoing | Mixed | | MDSAI | 3 |
| Peters ME et al  (2014; ^27^) | Breast, GI, Mixed | 137  (53/84) | Ongoing | Mixed, Chemo, Targeted, Endocrine | | CIS | 1 |
| Efficace F et al  (2015; ^28^) | Hematologic | 280  (176/104) | Before | Mixed | | EORTC QLQ-C30 | 2 |
| Poort H et al  (2016; ^29^) | GI | 89  (52/37) | Ongoing, After | Targeted, Surgery | | CIS | 1 |
| Lai JS et al  (2016; ^30^) | Mixed | 142  (84/58) | Present | Chemo | | Others | 1 |
| Lorca LA et al  (2016; ^31^) | Hematologic | 122  (61/61) | Present | Mixed | | BFI | 3 |
| Behringer K et al  (2016; ^32^) | Hematologic | 3619  (1980/1639) | Ongoing | Chemo | | EORTC-QLQ C30 | 1 |
| Poort H et al  (2017; ^33^) | Urologic, Mixed | 83  (43/40) | Ongoing, After, Present | Mixed, Surgery, Chemo, Radio | | CIS | 1 |
| Peoples AR et al  (2017; ^34^) | Breast | 548  (0/548) | Ongoing, Present | Mixed, Chemo | | BFI | 3, 1 |
| Alhashemi A et al  (2017; ^35^) | Head and neck | 203  (51/152) | Mixed | Surgery | | BFI | 3 |
| Kuhnt S et al  (2017; ^36^) | Mixed | 693  (300/393) | Mixed | Mixed | | MFI-20 | 3 |
| Pearce A et al  (2017; ^37^) | Mixed | 441  (115/326) | Ongoing | Chemo | | Others | 3 |
| Mao H et al  (2018; ^38^) | Breast | 1103  (0/1103) | Ongoing | Endocrine, Chemo, Radio, Surgery | | BFI | 3, 1 |
| Jung Jy et al  (2018; ^39^) | Lung | 830  (637/193) | Free | Mixed | | BFI | 3 |
| Smet S et al  (2018; ^40^) | Gynecologic | 1164  (0/1164) | Before, After | Mixed, Radio | | Others | 3 |
| Hofer F et al  (2018; ^41^) | Hematologic | 149  (80/69) | Before | None | | EORTC QLQ-C30 | 1 |
| Lacourt TE et al  (2018; ^42^) | Hematologic | 79  (41/38) | Before | None | | MDSAI | 2 |
| Bossi P et al  (2019; ^43^) | Head and neck | 129  (87/42) | Free | Radio | | BFI | 3 |
| Roila F et al  (2019; ^44^) | Mixed | 1394  (581/813) | Mixed | Mixed | | BFI | 2 |
| Rodríguez Antolín A et al  (2019; ^45^) | Urologic | 235  (235/0) | Present | Mixed, Endocrine | | BFI | 3 |
| Matias M et al  (2019; ^46^) | Breast, Urologic, GI, Mixed | 1984  (610/1374) | After | Mixed, Chemo, Surgery, Radio, Endocrine | | EORTC QLQ-C30 | 1 |
| Van der Linden SD et al  (2020; ^47^) | Brain | 65  (17/48) | Before, After | Surgery, Mixed | | MFI-20 | 2 |
| Poort H et al  (2020; ^48^) | Hematologic, Lung, Breast, Brain, Mixed | 180  (84/96) | Ongoing | Targeted, Chemo, Mixed | | BFI | 1 |
| Gernier F et al  (2020; ^49^) | Breast, Gynecologic, GI | 263  (48/215) | Free | Surgery | | MFI-20 | 2 |
| AlFayyad I et al  (2020; ^50^) | Hematologic | 168  (103/65) | Present | Mixed | | Others | 3 |
| Pearson EJ et al  (2021; ^51^) | Mixed | 411  (206/205) | Before, Ongoing, After | Mixed | | ESAS-r and Fatigue Pictogram | 2 |
| Ghaderi M et al  (2021; ^52^) | Mixed | 131  (72/59) | Free | Mixed | | BFI | 3 |
| van Deuren S et al  (2021; ^53^) | GI, Urologic, Mixed, Brain, Hematologic | 2810  (1464/1346) | Free | Mixed | | SFQ | 1 |
| Williams GR et al  (2021; ^54^) | GI | 364  (205/159) | Before | None | | PROMIS Global 10 | 3 |
| Maass SWMC et al  (2021; ^55^) | Breast | 350  (0/350) | Free | Mixed | | MFI-20 | 1 |
| Iguchi T et al  (2021; ^56^) | Prostate | 22  (22/0) | Ongoing | Endocrine | | BFI, Symptoms Report | 3 |
| Sedighi Pashaki A et al  (2021; ^57^) | Breast | 74  (0/74) | Ongoing | Radio | | BFI | 1 |
| ^a^ Single level (‘severe’ or ‘moderate to severe’ or ‘strong’, ‘strong’ was considered as ‘severe’), 2 levels (‘moderate’ *vs.* ‘severe’ or ‘mild’ *vs.* ‘moderate to severe’), or 3 levels (‘mild’ *vs.* ‘moderate’ *vs.* ‘severe’). | | | | | | | |

**References**

1. Stone P, Hardy J, Broadley K, et al: Fatigue in advanced cancer: a prospective controlled cross-sectional study. British journal of cancer 79:1479-1486, 1999

2. Stone P, Richards M, A’hern R, et al: A study to investigate the prevalence, severity and correlates of fatigue among patients with cancer in comparison with a control group of volunteers without cancer. Annals of oncology 11:561-568, 2000

3. Stone P, Hardy J, Huddart R, et al: Fatigue in patients with prostate cancer receiving hormone therapy. European Journal of Cancer 36:1134-1141, 2000

4. Stone P, Richards M, A'Hern R, et al: Fatigue in patients with cancers of the breast or prostate undergoing radical radiotherapy. Journal of pain and symptom management 22:1007-1015, 2001

5. Wang XS, Giralt SA, Mendoza TR, et al: Clinical factors associated with cancer-related fatigue in patients being treated for leukemia and non-Hodgkin’s lymphoma. Journal of Clinical Oncology 20:1319-1328, 2002

6. De Jong N, Candel M, Schouten H, et al: Prevalence and course of fatigue in breast cancer patients receiving adjuvant chemotherapy. Annals of oncology 15:896-905, 2004

7. Hickok JT, Roscoe JA, Morrow GR, et al: Frequency, severity, clinical course, and correlates of fatigue in 372 patients during 5 weeks of radiotherapy for cancer. Cancer 104:1772-1778, 2005

8. Meeske K, Smith AW, Alfano CM, et al: Fatigue in breast cancer survivors two to five years post diagnosis: a HEAL Study report. Quality of Life Research 16:947-960, 2007

9. Storey D, Waters RA, Hibberd CJ, et al: Clinically relevant fatigue in cancer outpatients: the Edinburgh Cancer Centre symptom study. Annals of oncology 18:1861-1869, 2007

10. Gielissen M, Schattenberg A, Verhagen C, et al: Experience of severe fatigue in long-term survivors of stem cell transplantation. Bone marrow transplantation 39:595-603, 2007

11. Servaes P, Gielissen M, Verhagen S, et al: The course of severe fatigue in disease‐free breast cancer patients: a longitudinal study. Psycho‐Oncology: Journal of the Psychological, Social and Behavioral Dimensions of Cancer 16:787-795, 2007

12. Kim SH, Son BH, Hwang SY, et al: Fatigue and depression in disease-free breast cancer survivors: prevalence, correlates, and association with quality of life. Journal of pain and symptom management 35:644-655, 2008

13. Goedendorp M, Gielissen M, Verhagen C, et al: Severe fatigue and related factors in cancer patients before the initiation of treatment. British journal of cancer 99:1408-1414, 2008

14. Kuhnt S, Ernst J, Singer S, et al: Fatigue in cancer survivors–prevalence and correlates. Oncology Research and Treatment 32:312-317, 2009

15. Huang X, Zhang Q, Kang X, et al: Factors associated with cancer-related fatigue in breast cancer patients undergoing endocrine therapy in an urban setting: a cross-sectional study. BMC cancer 10:1-7, 2010

16. Prigozin A, Uziely B, Musgrave CF: The relationship between symptom severity and symptom interference, education, age, marital status, and type of chemotherapy treatment in Israeli women with early-stage breast cancer, Oncology Nursing Forum, 2010

17. Lamino DdA, Mota DDCdF, Pimenta CAdM: Prevalence and comorbidity of pain and fatigue in women with breast cancer. Revista da Escola de Enfermagem da USP 45:508-514, 2011

18. Hung R, Krebs P, Coups EJ, et al: Fatigue and functional impairment in early-stage non-small cell lung cancer survivors. Journal of pain and symptom management 41:426-435, 2011

19. Liu Y, Xi Q-s, Xia S, et al: Association between symptoms and their severity with survival time in hospitalized patients with far advanced cancer. Palliative Medicine 25:682-690, 2011

20. Li Y, Yuan C: Levels of fatigue in Chinese women with breast cancer and its correlates: A cross‐sectional questionnaire survey. Journal of the American Academy of Nurse Practitioners 23:153-160, 2011

21. Karthikeyan G, Jumnani D, Prabhu R, et al: Prevalence of fatigue among cancer patients receiving various anticancer therapies and its impact on quality of life: a cross-sectional study. Indian Journal of Palliative Care 18:165, 2012

22. Mota DDCdF, Pimenta CAdM, Caponero R: Fatigue in colorectal cancer patients: prevalence and associated factors. Revista latino-americana de enfermagem 20:495-503, 2012

23. Goedendorp MM, Gielissen MF, Verhagen CA, et al: Development of fatigue in cancer survivors: a prospective follow-up study from diagnosis into the year after treatment. Journal of pain and symptom management 45:213-222, 2013

24. Tian J, Hong J-S: Application of the Chinese version of the MFI-20 in detecting the severe fatigue in cancer patients. Supportive Care in Cancer 21:2217-2223, 2013

25. Cleeland CS, Zhao F, Chang VT, et al: The symptom burden of cancer: evidence for a core set of cancer‐related and treatment‐related symptoms from the Eastern Cooperative Oncology Group Symptom Outcomes and Practice Patterns study. Cancer 119:4333-4340, 2013

26. Wang XS, Zhao F, Fisch MJ, et al: Prevalence and characteristics of moderate to severe fatigue: a multicenter study in cancer patients and survivors. Cancer 120:425-432, 2014

27. Peters ME, Goedendorp MM, Verhagen CA, et al: Severe fatigue during the palliative treatment phase of cancer: an exploratory study. Cancer Nursing 37:139-145, 2014

28. Efficace F, Gaidano G, Breccia M, et al: Prevalence, severity and correlates of fatigue in newly diagnosed patients with myelodysplastic syndromes. British journal of haematology 168:361-370, 2015

29. Poort H, van der Graaf WT, Tielen R, et al: Prevalence, impact, and correlates of severe fatigue in patients with gastrointestinal stromal tumors. Journal of Pain and Symptom Management 52:265-271, 2016

30. Lai J-S, Haertling T, Weinstein J, et al: A cross-sectional study of carnitine deficiency and fatigue in pediatric cancer patients. Child's Nervous System 32:475-483, 2016

31. Lorca LA, Sacomori C, Puga B: Assessment of a brief fatigue inventory in patients with hematologic malignancies. Revista Medica de Chile 144:894-899, 2016

32. Behringer K, Goergen H, Müller H, et al: Cancer-related fatigue in patients with and survivors of Hodgkin lymphoma: the impact on treatment outcome and social reintegration. Journal of Clinical Oncology 34:4329-4337, 2016

33. Poort H, Kaal SE, Knoop H, et al: Prevalence and impact of severe fatigue in adolescent and young adult cancer patients in comparison with population-based controls. Supportive Care in Cancer 25:2911-2918, 2017

34. Peoples AR, Roscoe JA, Block RC, et al: Nausea and disturbed sleep as predictors of cancer-related fatigue in breast cancer patients: a multicenter NCORP study. Supportive Care in Cancer 25:1271-1278, 2017

35. Alhashemi A, Jones JM, Goldstein DP, et al: An exploratory study of fatigue and physical activity in Canadian thyroid cancer patients. Thyroid 27:1156-1163, 2017

36. Kuhnt S, Szalai C, Erdmann-Reusch B, et al: Cancer related fatigue in rehabilitation care. Die Rehabilitation 56:337-343, 2017

37. Pearce A, Haas M, Viney R, et al: Incidence and severity of self-reported chemotherapy side effects in routine care: A prospective cohort study. PloS one 12:e0184360, 2017

38. Mao H, Bao T, Shen X, et al: Prevalence and risk factors for fatigue among breast cancer survivors on aromatase inhibitors. European Journal of Cancer 101:47-54, 2018

39. Jung JY, Lee JM, Kim MS, et al: Comparison of fatigue, depression, and anxiety as factors affecting posttreatment health‐related quality of life in lung cancer survivors. Psycho‐Oncology 27:465-470, 2018

40. Smet S, Pötter R, Haie-Meder C, et al: Fatigue, insomnia and hot flashes after definitive radiochemotherapy and image-guided adaptive brachytherapy for locally advanced cervical cancer: an analysis from the EMBRACE study. Radiotherapy and oncology 127:440-448, 2018

41. Hofer F, Koinig K, Nagl L, et al: Fatigue at baseline is associated with geriatric impairments and represents an adverse prognostic factor in older patients with a hematological malignancy. Annals of hematology 97:2235-2243, 2018

42. Lacourt TE, Kavelaars A, Ohanian M, et al: Patient–reported fatigue prior to treatment is prognostic of survival in patients with acute myeloid leukemia. Oncotarget 9:31244, 2018

43. Bossi P, Di Pede P, Guglielmo M, et al: Prevalence of fatigue in head and neck cancer survivors. Annals of Otology, Rhinology & Laryngology 128:413-419, 2019

44. Roila F, Fumi G, Ruggeri B, et al: Prevalence, characteristics, and treatment of fatigue in oncological cancer patients in Italy: a cross-sectional study of the Italian Network for Supportive Care in Cancer (NICSO). Supportive Care in Cancer 27:1041-1047, 2019

45. Rodríguez Antolín A, Martínez-Piñeiro L, Jiménez Romero M, et al: Prevalence of fatigue and impact on quality of life in castration-resistant prostate cancer patients: The VITAL study. BMC urology 19:1-8, 2019

46. Matias M, Baciarello G, Neji M, et al: Fatigue and physical activity in cancer survivors: A cross‐sectional population‐based study. Cancer medicine 8:2535-2544, 2019

47. van der Linden SD, Gehring K, Rutten G-JM, et al: Prevalence and correlates of fatigue in patients with meningioma before and after surgery. Neuro-Oncology Practice 7:77-85, 2020

48. Poort H, Jacobs JM, Pirl WF, et al: Fatigue in patients on oral targeted or chemotherapy for cancer and associations with anxiety, depression, and quality of life. Palliative & supportive care 18:141-147, 2020

49. Gernier F, Joly F, Klein D, et al: Cancer-related fatigue among long-term survivors of breast, cervical, and colorectal cancer: a French registry–based controlled study. Supportive Care in Cancer 28:5839-5849, 2020

50. AlFayyad I, Al-Tannir M, Yaqub M, et al: Clinically Significant Fatigue in Adult Leukemia Patients: Prevalence, Predictors, and Impact on Quality of Life. Cureus 12, 2020

51. Pearson EJ, Drosdowsky A, Edbrooke L, et al: Exploring the Use of Two Brief Fatigue Screening Tools in Cancer Outpatient Clinics. Integrative cancer therapies 20:1534735420983443, 2021

52. Ghaderi M, Azadi A, Rahmani A, et al: Fatigue and Its Related Factors Among Iranian Cancer Survivors. Journal of caring sciences 10:210, 2021

53. van Deuren S, Penson A, van Dulmen‐den Broeder E, et al: Prevalence and risk factors of cancer‐related fatigue in childhood cancer survivors: A DCCSS LATER study. Cancer 128:1110-1121, 2022

54. Williams GR, Al-Obaidi M, Dai C, et al: Fatigue is independently associated with functional status limitations in older adults with gastrointestinal malignancies—results from the CARE registry. Supportive Care in Cancer 29:6793-6800, 2021

55. Maass SW, Brandenbarg D, Boerman LM, et al: Fatigue among long-term breast cancer survivors: a controlled cross-sectional study. Cancers 13:1301, 2021

56. Iguchi T, Nakamura Y, Akiyama T, et al: Descriptive study on burden and communication of fatigue among castration-resistant prostate cancer patients in Japan. Current Medical Research and Opinion 38:417-426, 2022

57. Sedighi Pashaki A, Mohammadian K, Afshar S, et al: A randomized, controlled, parallel-group, trial on the effects of melatonin on fatigue associated with breast cancer and its adjuvant treatments. Integrative Cancer Therapies 20:1534735420988343, 2021
