# Supplementary material for: Prevalence of cancer-related fatigue based on severity: a systematic review and meta-analysis
Source: Sci Rep. 2023 Aug 7;13:12815. doi: 10.1038/s41598-023-39046-0 (PMC10406927; doi:10.1038/s41598-023-39046-0)
Supplement: Supplementary file 2 — Supplementary Legends. [file 41598_2023_39046_MOESM2_ESM.doc]

**Legend for Suppl. Fig 1.**

After removing the 45 data of patients undergoing treatment, meta-analysis-derived prevalence of CRF according to 4 levels of severity and 8 organ/system types of cancer origin are presented inside each circle, and the prevalence rates are proportional to the size of the circles. The 95% CI is displayed only for the ‘total’ prevalence, which was synthesized with prevalence indicated as fatigue per data, regardless of severity. ^a^ indicates the number of data used for meta-analysis. ^b^ indicates the number of patients enrolled for data analysis (some participants were counted repeatedly for mixed cancer).

**Legend for Suppl. Fig 2.**

A graphic summary depicting the overall prevalence of CRF was created, illustrating the distribution based on severity, cancer phase, and gender, respectively.
