# Supplementary material for: Prevalence of cancer-related fatigue based on severity: a systematic review and meta-analysis
Source: Sci Rep. 2023 Aug 7;13:12815. doi: 10.1038/s41598-023-39046-0 (PMC10406927; doi:10.1038/s41598-023-39046-0)
Supplement: Supplementary file 3 — Supplementary Figure 1. [file 41598_2023_39046_MOESM3_ESM.pptx]

## Slide 1
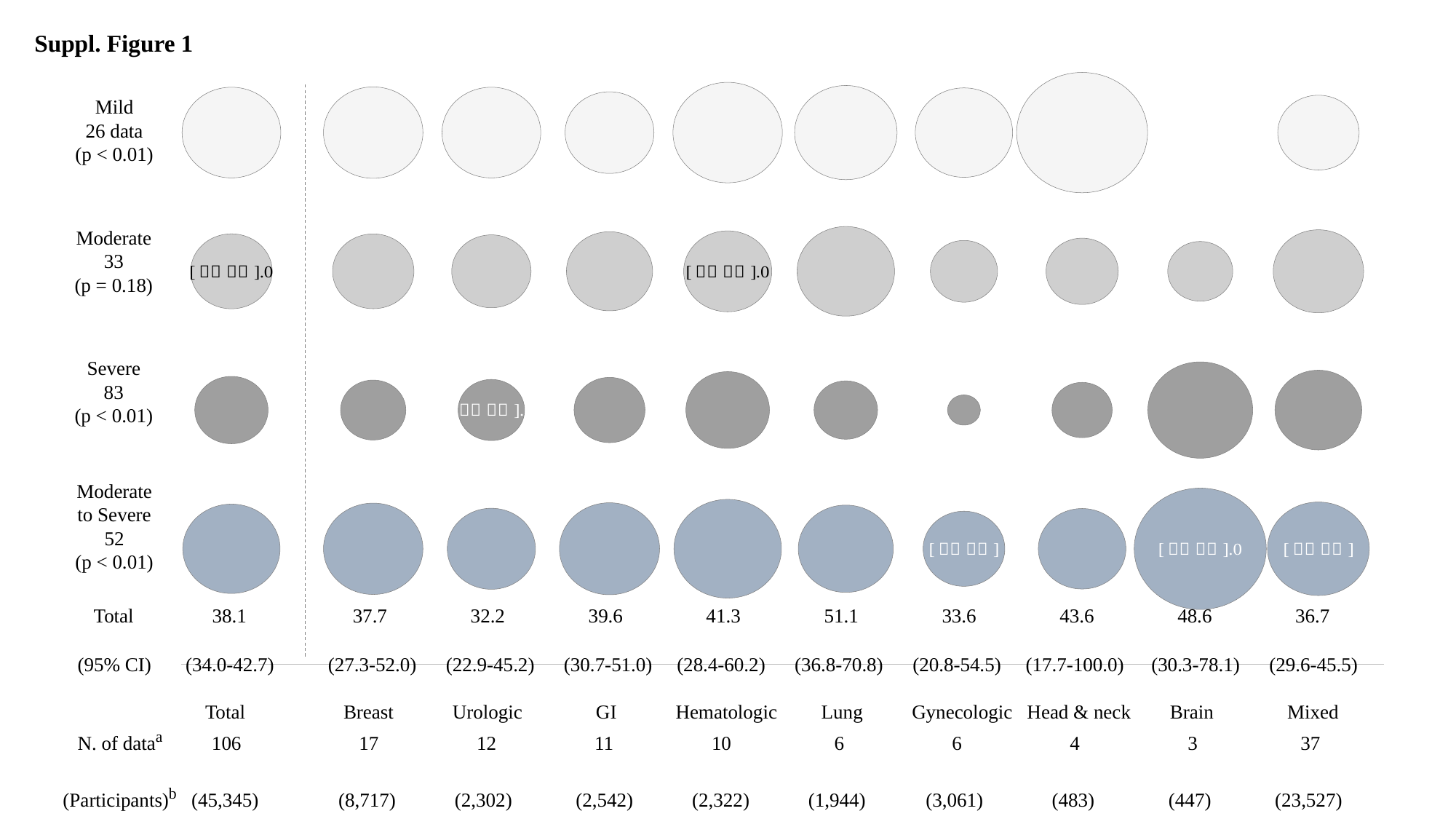

Suppl. Figure 1
### Chart
| Category | Y 값 |
|---|---| Total 38.1 37.7 32.2 39.6 41.3 51.1 33.6 43.6 48.6 36.7
 (95% CI) (34.0-42.7) (27.3-52.0) (22.9-45.2) (30.7-51.0) (28.4-60.2) (36.8-70.8) (20.8-54.5) (17.7-100.0) (30.3-78.1) (29.6-45.5)
 Total Breast Urologic GI Hematologic Lung Gynecologic Head & neck Brain Mixed
 N. of dataa 106 17 12 11 10 6 6 4 3 37
(Participants)b (45,345) (8,717) (2,302) (2,542) (2,322) (1,944) (3,061) (483) (447) (23,527)
Mild
26 data
(p < 0.01)
Moderate
33
(p = 0.18)
Severe
83
(p < 0.01)
Moderate
to Severe
52
(p < 0.01)
