# Supplementary material for: Prevalence of cancer-related fatigue based on severity: a systematic review and meta-analysis
Source: Sci Rep. 2023 Aug 7;13:12815. doi: 10.1038/s41598-023-39046-0 (PMC10406927; doi:10.1038/s41598-023-39046-0)
Supplement: Supplementary file 4 — Supplementary Figure 2. [file 41598_2023_39046_MOESM4_ESM.pptx]

## Slide 1
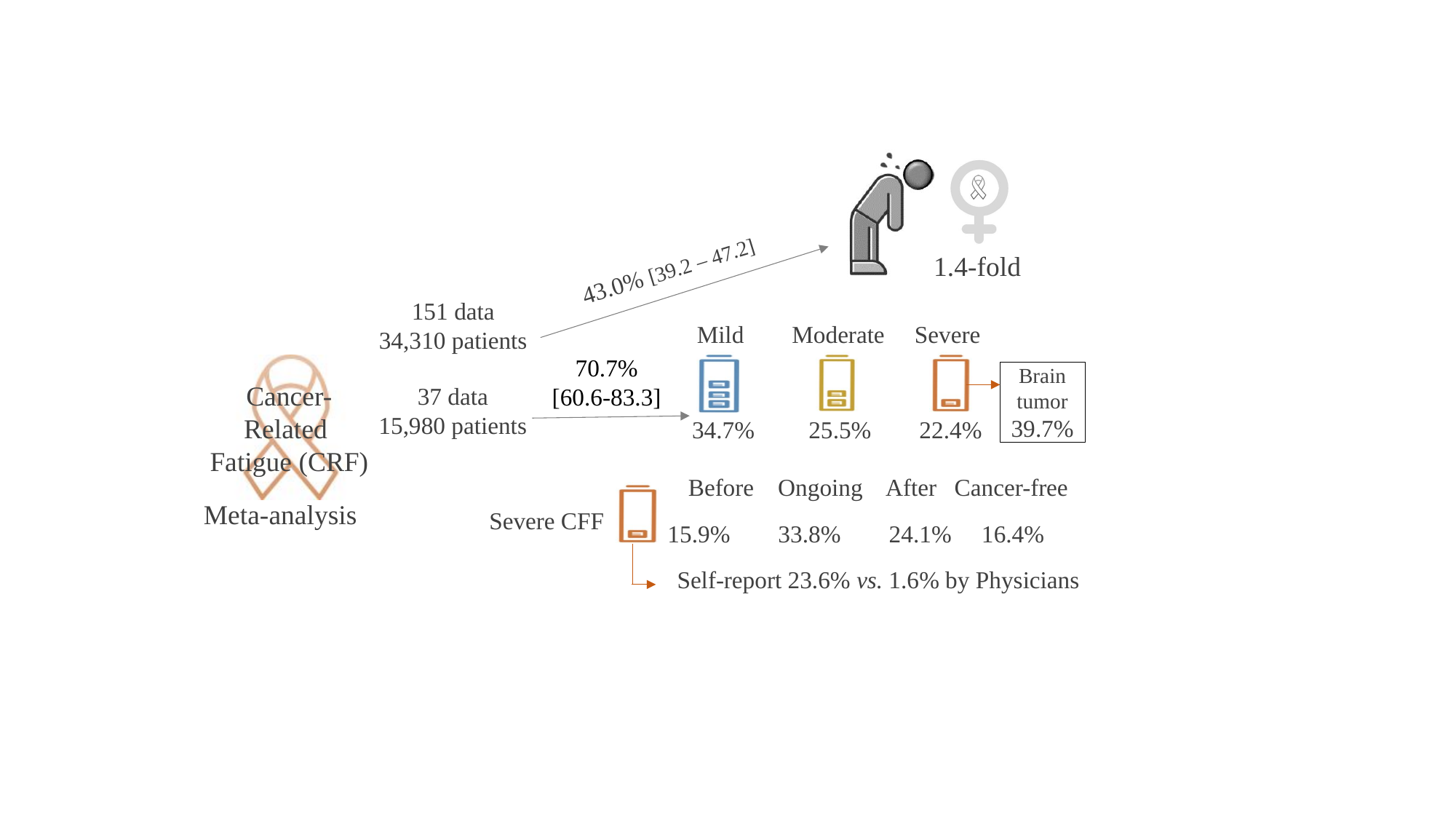

1.4-fold
43.0% [39.2 – 47.2]
151 data
34,310 patients
 Mild Moderate Severe
70.7%
[60.6-83.3]
Cancer-
Related
Fatigue (CRF)
Meta-analysis
Brain tumor 39.7%
37 data
15,980 patients
34.7% 25.5% 22.4%
 Before Ongoing After Cancer-free
Severe CFF
15.9% 33.8% 24.1% 16.4%
 Self-report 23.6% vs. 1.6% by Physicians
